# Chagas Disease Risk in Texas

**DOI:** 10.1371/journal.pntd.0000836

**Published:** 2010-10-05

**Authors:** Sahotra Sarkar, Stavana E. Strutz, David M. Frank, Chissa–Louise Rivaldi, Blake Sissel, Victor Sánchez–Cordero

**Affiliations:** 1 Section of Integrative Biology, University of Texas, Austin, Texas, United States of America; 2 Department of Philosophy, University of Texas, Austin, Texas, United States of America; 3 Instituto de Biología, Universidad Nacional Autónoma de México, México City, México; Lancaster University, United Kingdom

## Abstract

**Background:**

Chagas disease, caused by *Trypanosoma cruzi*, remains a serious public health concern in many areas of Latin America, including México. It is also endemic in Texas with an autochthonous canine cycle, abundant vectors (Triatoma species) in many counties, and established domestic and peridomestic cycles which make competent reservoirs available throughout the state. Yet, Chagas disease is not reportable in Texas, blood donor screening is not mandatory, and the serological profiles of human and canine populations remain unknown. The purpose of this analysis was to provide a formal risk assessment, including risk maps, which recommends the removal of these lacunae.

**Methods and Findings:**

The spatial relative risk of the establishment of autochthonous Chagas disease cycles in Texas was assessed using a five–stage analysis. 1. Ecological risk for Chagas disease was established at a fine spatial resolution using a maximum entropy algorithm that takes as input occurrence points of vectors and environmental layers. The analysis was restricted to triatomine vector species for which new data were generated through field collection and through collation of post–1960 museum records in both México and the United States with sufficiently low georeferenced error to be admissible given the spatial resolution of the analysis (1 arc–minute). The new data extended the distribution of vector species to 10 new Texas counties. The models predicted that *Triatoma gerstaeckeri* has a large region of contiguous suitable habitat in the southern United States and México, *T. lecticularia* has a diffuse suitable habitat distribution along both coasts of the same region, and T. *sanguisuga* has a disjoint suitable habitat distribution along the coasts of the United States. The ecological risk is highest in south Texas. 2. Incidence–based relative risk was computed at the county level using the Bayesian Besag–York–Mollié model and post–1960 *T. cruzi* incidence data. This risk is concentrated in south Texas. 3. The ecological and incidence–based risks were analyzed together in a multi–criteria dominance analysis of all counties and those counties in which there were as yet no reports of parasite incidence. Both analyses picked out counties in south Texas as those at highest risk. 4. As an alternative to the multi–criteria analysis, the ecological and incidence–based risks were compounded in a multiplicative composite risk model. Counties in south Texas emerged as those with the highest risk. 5. Risk as the relative expected exposure rate was computed using a multiplicative model for the composite risk and a scaled population county map for Texas. Counties with highest risk were those in south Texas and a few counties with high human populations in north, east, and central Texas showing that, though Chagas disease risk is concentrated in south Texas, it is not restricted to it.

**Conclusions:**

For all of Texas, Chagas disease should be designated as reportable, as it is in Arizona and Massachusetts. At least for south Texas, lower than 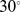
N, blood donor screening should be mandatory, and the serological profiles of human and canine populations should be established. It is also recommended that a joint initiative be undertaken by the United States and México to combat Chagas disease in the trans–border region. The methodology developed for this analysis can be easily exported to other geographical and disease contexts in which risk assessment is of potential value.

## Author Summary

Chagas disease is endemic in Texas and spread through triatomine insect vectors known as kissing bugs, assassin bugs, or cone–nosed bugs, which transmit the protozoan parasite, *Trypanosoma cruzi*. We examined the threat of Chagas disease due to the three most prevalent vector species and from human case occurrences and human population data at the county level. We modeled the distribution of each vector species using occurrence data from México and the United States and environmental variables. We then computed the ecological risk from the distribution models and combined it with disease incidence data to produce a composite risk map which was subsequently used to calculate the populations expected to be at risk for the disease. South Texas had the highest relative risk. We recommend mandatory reporting of Chagas disease in Texas, testing of blood donations in high risk counties, human and canine testing for Chagas disease antibodies in high risk counties, and that a joint initiative be developed between the United States and México to combat Chagas disease.

## Introduction

Chagas disease, a result of infection by the hemoflagellate kinetoplastid protozoan, *Trypanosoma cruzi*, remains an important public health threat in Latin America [1] with an estimated 16–18 million human incidences and 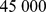 deaths annually [2]. While the Southern Cone Initiative [3]–[6] has interrupted the transmission of Chagas disease in several South American countries, and similar efforts are being attempted for other countries of Latin America [5]–[7], the disease is also endemic in the southern United States, especially in Texas where it is yet to be designated as reportable [8]–[13]. Moreover, patterns of human migration into Texas from endemic regions of Latin America may contribute to an increase in the risk of Chagas disease [11], [14], [15]. Because the disease has a chronic phase that may last for decades, during which parasitaemia falls to undetectable levels [7], the extent of human infection in the southern United States is at present unknown. Based entirely on demographics, Hanford *et al.* [10] provided an extreme estimate of more than 1 million infections for the United States with 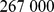 of them being in Texas. However, Bern and Montgomery [11] have criticized that estimate for using the highest possible values for all contributory factors; they provide a more credible lower estimate of 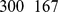 for the entire United States. Infections of zoonotic origin only add to the number of infections of demographic origin and the risk of disease. So far infected vectors or hosts have been found in 82 of the 254 counties of Texas (see Table S1) though only four vector–borne human autochthonous cases have been confirmed [16]. The parasite incidence rate in vectors in Texas has been reported as being 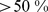 [12], [16], [17] which is higher than the 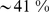 reported from Phoenix, Arizona [13], but lower than the 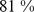 reported from Guaymas in northwestern México [18]. In contrast to Texas, the disease is reportable in Arizona and Massachusetts even though there has not been an autochthonous human case in either state, compared to the four in Texas. The other autochthonous human cases confirmed for the United States are from California [19], Tennessee [20], and Louisiana [9].

The main human Chagas disease cycle consists of the parasite, *T. cruzi*, being transferred from a mammalian reservoir to a human host through a vector. However, infection through blood transfusion, organ transplants, and the ingestion of infected food are also recognized mechanisms of concern; infections may also occur through congenital transmission [7], [21], [22]. A large variety of mammal species can serve as reservoirs for *T. cruzi* including humans and dogs [7], which means that a focus on reservoirs would not be effective for disease control. Given that no vaccine exists [23], efforts to control the disease must focus on vector control [7]. Consequently, risk assessment for Chagas disease must focus primarily on the ecology and biogeography of vector species and the incidence of the parasite, besides human social and epidemiological factors [5].

This analysis consists of a five–stage risk assessment for Chagas disease in Texas: (i) an ecological risk analysis using predicted vector distributions; (ii) an incidence–based risk analysis based on parasite occurrence; (iii) a joint analysis of ecology and incidence using formal multi–criteria analysis; (iv) such a joint analysis using a composite risk model; and (v) a computation of the relative expected exposure rate taking into account human population. The purpose of the complete analysis is to argue that there is sufficient widespread risk for Chagas disease in Texas to warrant it to be declared reportable and other measures be taken. The analysis focuses primarily on the vector distributions but also uses available information on parasite incidence. If the number of human infections in Texas is as high as in the estimates noted earlier [10], [11], then humans alone would constitute sufficient reservoirs in disease foci. Moreover, even if the number of human infections is much lower, there is compelling evidence that the disease has established itself in Texas in domestic and peridomestic cycles with canine reservoirs [16], [17]. Thus, also given the abundance of wild zoonotic reservoirs in most of the state, including armadillos, coyotes, raccoons, opossums, and rodents of the genus Neotoma, the distribution of reservoirs is not likely to limit the occurrence or spread of the disease in Texas. This analysis assumes that competent reservoirs are present everywhere in Texas in sufficient densities to perpetuate or establish the disease cycle. Moreover, the peridomestic cycle makes human exposure to the parasite more likely than what would have been the case with only a sylvatic transmission cycle.

The vectors of Chagas disease are insects from the family Reduviidae, sub–family Triatominae, and in northern México and the United States, restricted to the genus Triatoma. Seven Triatoma species have been routinely collected in Texas: *Triatoma gerstaeckeri*, *T. sanguisuga*, *T. lecticularia*, *T. protracta*, *T. indictiva*, *T. rubida*, and *T. neotomae* [12]. (One specimen of *T. recurva* was reported from Brewster county in far southwestern Texas on the Mexican border in 1984 [24] but no further specimen has since been found in Texas; available records are restricted to Arizona and northwestern México.)

Using data from new field collections as well as museum records, this analysis begins by constructing species distribution models for the three most widely distributed Triatoma species in Texas: *T. gerstaeckeri*, *T. sanguisuga*, and *T. lecticularia*. All three species have been shown to be carriers of *T. cruzi* [12], [25]. The other four Triatoma species were so rare (collected less than 10 times in total by any researcher in Texas since 2000) that they are presumed not to be important for establishing Chagas disease transmission cycles in the state. The species distribution models were constructed using a maximum entropy algorithm which relies on species occurrence (presence–only) records and environmental layers [26]. Such a modeling strategy, though using a genetic algorithm, has been previously used to model the distribution of *T. gerstaeckeri* in Texas [16], and a variety of triatomine species complexes for North America [27] though at a much coarser spatial resolution than this analysis which used cells with 1 arc-minute edges. The output from these models directly quantify habitat suitability for a species by computing the relative probability of its presence in each cell of the study area. These probabilities establish the potential distribution of a species (and are sometimes interpreted as providing an approximate ecological niche model [28], [29]). The predicted distribution is obtained using biological information such as dispersal behavior and other constraints that limit the potential distribution.

These three species' distributions were used to generate a map of the probability of the occurrence of at least one triatomine vector species in each cell. This is the most basic ecological risk map: when these probabilities are low, there is little risk of Chagas disease occurrence through the major vectorial mode of transmission though disease may still occur through contaminated blood transfusion and, less likely, through parasite ingestion. (By “risk,” throughout this paper, we will mean *relative risk*, that is, the risk in one cell compared to others throughout the area of interest.) When the ecological (relative) risk is high, other risk factors determine the likelihood of disease, including the abundance of vectors, the incidence of parasites, and anthropogenic features of the habitat, for instance, human behavioral patterns (including habitation structure) [30], [31]. Ecological risk maps of this kind have previously been used for this region to estimate the risk of the spread of leishmaniasis due to climate change [31]. The relevance of that work to the present analysis is that the disease agents for leishmaniasis are also kinetoplastid protozoans which share reservoirs with *T. cruzi* [32]–[36].

Independently, at the county level (which was the finest resolution at which data were available), a (relative) risk map based on parasite incidence in vectors, canine reservoirs, or humans was constructed using the Bayesian Besag-York-Mollié (BYM) model which is widely used in epidemiology [37]. This map was based on a spatial interpolation of risk from the number of parasite records from each county: it captures the idea that there is spatial correlation between disease incidences. The implications of the incidence–based risk map were combined with those of the basic ecological risk map in two ways: (i) a simple multi-criteria analysis (MCA) [38] was used to find the counties that were most at risk from both suitability for vector species and proximity to locations of parasite incidence; (ii) a multiplicative risk model was used to obtain a composite risk map for Chagas disease in Texas. Both sets of results were used to prioritize counties for increased surveillance for the occurrence of *T. cruzi*.

Finally, the composite risk map was combined with the relative human population densities of the counties to produce a “relative expected exposure rate” risk map which provides a rough relative measure of potential extent of human exposure to Chagas disease. The entire risk analysis was used to recommend that Chagas disease be made reportable in Texas, that the blood supply be screened in south Texas, and that human and canine serological profiles be investigated in the same region.

## Materials and Methods

### Study Area

**Figure 1:**
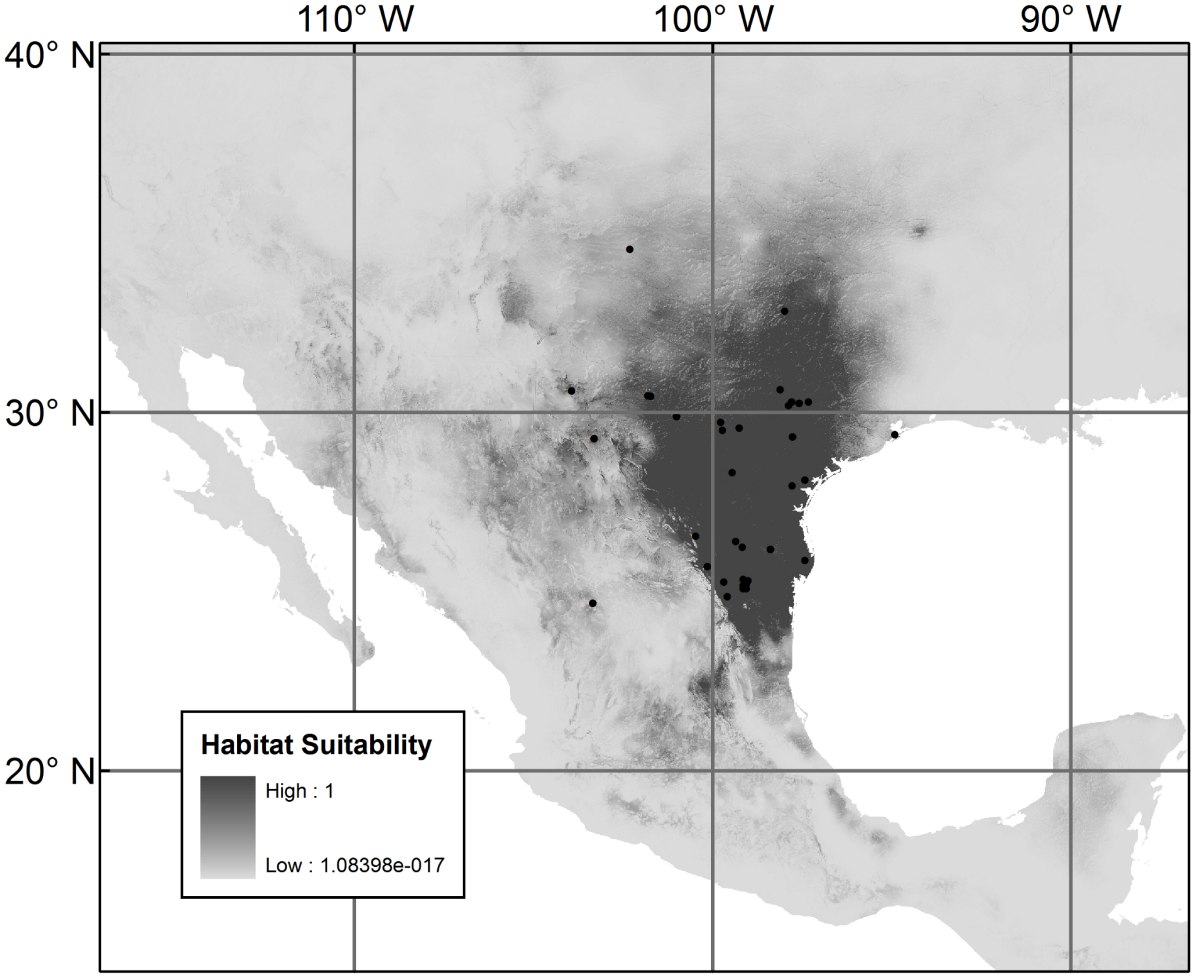
Species distribution model for *Triatoma gerstaeckeri*. The black dots show the occurrence points used for model construction.

The study area was delimited at the south by the 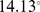
N line of latitude along the México-Guatemala border, by the coast of continental México to the east and west, continued by the lines 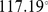
W and 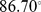
W within the United States and the line 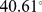
N at the north, thus enclosing all the species' occurrence points (see Figure 1). It was divided into 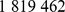 cells at a resolution of 1 arc–minute. The average cell area was 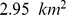.

### Species Distribution Models

Species distribution models were constructed for the three most important triatomine vector species in Texas [12]: *T. gerstaeckeri*, *T. lecticularia*, and *T. sanguisuga*.

#### Data

Triatomine species occurrence data were obtained from museum collections, other researchers, voluntary collectors (see Acknowledgments for more detailed information on all three categories), as well as organized surveys in Texas and northern México, the results of which will be reported separately in the ecological literature. Species were identified using the key of Lent and Wygodzinsky [39]. All data were entered in the Disease Vectors Database (www.diseasevectors.org; last accessed 28 February 2010; [40]) and were georeferenced using the MaNIS protocol (http://manisnet.org/GeorefGuide.html; last accessed 28 February 2010) which has been extensively developed and refined by ecologists for this purpose. (Table S2 shows the number of records that were available for each species.)

For modeling purposes, because of the spatial resolution of the analysis, only records with an estimated error less than 1 arc–minute were retained. Moreover, because the WorldClim environmental layers only average information since 1960, all pre–1960 records were excluded from this analysis. With one exception for *T. lecticularia* and two exceptions for *T. sanguisuga*, all records were post–1980. There were 74 records retained for *T. gerstaeckeri*, 23 for *T. sanguisuga*, and 11 for *T. lecticularia*; these records generated 35, 17, and 11 instances in different cells, respectively, at the spatial resolution of this analysis.

Because only post–1960 triatomine records were used for the species distribution models, parasite incidence records used in this analysis were also restricted to the same period. *T. cruzi* incidence data in Triatoma, canines, and humans were compiled from the literature using the citations of recent reviews [10], [12], [17] through a backward search of earlier reports until 1960. Records of parasite incidence in vectors and human and canine hosts were used; there was little reliable information on other hosts. (These data are summarized in Table S1.)

Human population data per county were obtained from the Texas State Data Center and Office of the State Demographer (http://txsdc.utsa.edu/tpepp/2008_txpopest_county.php; last accessed 4-March-2010). July 2008 population estimate data were used; these are the most recent estimates available for every county and are based on the 2000 census. Economic data for these counties were obtained from the United States Census Bureau [41].

#### Model Construction

The species distribution models were constructed from species' occurrence points and environmental layers using a maximum entropy algorithm. The Maxent software package (Version 3.3.4; [26]) was used to construct the models. Maxent has been shown to be robust for modeling species distributions from occurrence (presence–only) records for a large number of taxa [42]. Following published recommendations [26], [43], [44], Maxent was run with the threshold and hinge features and without duplicates so that there was at most one sample per pixel; linear, quadratic, and product features were used. The convergence threshold was set to a conservative 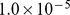. For the AUC, that is, the area under the receiver operating characteristic (ROC) curve, averages over 100 replicate models were computed. For each model the test∶training ratio was set to 40∶60 following Phillips and Dudík [26] which means that models were constructed using 60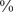 of the data and tested with the remaining 40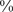.

Two tests were used to assess model performance: (i) A conservative threshold of 0.9 was used for the test AUC. (An optimal model would have an AUC close to 1 while a model that predicted species occurrences at random would have an AUC of 0.5. Published recommendations suggest using a minimum threshold of 0.7 [42].); (ii) For the eight internal training and test binomial tests performed by Maxent (two each for minimum presence, 10 percentile presence, equal sensitivity and specificity, maximum sensitivity plus specificity), on the average, a *p*-value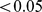 was required.

The environmental layers used are listed in Table 1. These include four topographical variables (elevation, slope, aspect, and composite topographical index) and 15 bioclimatic variables. The latter were obtained from the WorldClim database (www.worldclim.org; last accessed 28 February 2010; [45]). However, of the standard 19 bioclimatic variables, four were excluded (mean temperatures of the wettest quarter, driest quarter, warmest quarter, and coldest quarter) because the layers contain discontinuities within the study area from Texas. These discontinuities seem to be artefacts introduced during the interpolation used to construct the layers. Elevation was obtained from the United States Geological Survey's Hydro–1K DEM data set (http://eros.usgs.gov/#/Find_Data/Products_and_Data_Available/gtopo30/hydro; last accessed 28 February 2010). Slope, aspect, and compound topographical index were derived from the DEM using the Spatial Analyst extension of ArcMap 9.3.

**Table 1:** Environmental parameters for species distribution models.

Parameters
Annual Mean Temperature
Mean Diurnal Range
Isothermality
Temperature Seasonality
Maximum Temperature of Warmest Month
Minimum Temperature of Coldest Month
Temperature Annual Range
Annual Precipitation
Precipitation of Wettest Month
Precipitation of Driest Month
Precipitation Seasonality
Precipitation of Wettest Quarter
Precipitation of Driest Quarter
Precipitation of Warmest Quarter
Precipitation of Coldest Quarter
Elevation
Slope
Aspect
Compound Topographic Index

The use of a large number of environmental variables raises the possibility of over–fitting a model due to correlations between the explanatory variables (even though the algorithm in Maxent is designed to counteract such correlations). One sign of such over–fitting is a much lower AUC for the test data compared to the AUC for the training data. To judge the potential occurrence of this problem for the species distribution models, a second set of “simpler” models was constructed using the four topographic variables and only seven bioclimatic variables: the annual mean temperature, mean diurnal range, maximum temperature of the warmest month, minimum temperature of the coldest month, annual precipitation, precipitation of the wettest month, and precipitation of the driest month, which are all known to be of general ecological relevance. All other model parameters were uniform between the two sets. For each species, and each replicate model, the difference between the training AUC and the test AUC was calculated under each modeling choice resulting in two sets of 100 values for each species, one corresponding to the use of 19 environmental variables and the other to the use of 11 environmental variables. These data were not normally distributed (Shapiro test, 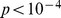). For each of the three pairs of 100 models, subsequent use of the Mann–Whitney-Wilcoxon test did not permit distinguishing the mean values of the AUC difference (minimum 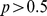). (All statistical computations were done in R.) Subsequently, models based on all 19 environmental variables were used for the rest of this analysis because they had higher test AUC values.

#### Probability of Triatomine Presence

The output from Maxent consists of relative suitability values between 0 and 1 which, when normalized, can be interpreted as the probability of occurrence of a species in a landscape cell. The probability that at least one triatomine species is present in a cell was computed as the complement of the probability that none is present. This computation assumed that the probability of the presence of each species is independent of that of the presence of another species. This assumption is reasonable because different species are often found at the same location and there is no evidence of competitive or other interactions between them [27].

Let the probability of the presence of at least one triatomine species in cell 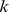 be 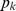 and that of species 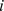 in cell 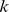 be 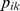. Then:
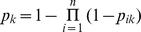
where 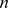 is the number of species. In this case there were three species, *T. gerstaeckeri*, *T. sanguisuga*, and *T. lecticularia*.

### Risk Assessment

#### Components of Risk

The concept of risk is salient only in those circumstances in which there is a chance of some undesirable event happening. Consequently, two broad components of risk can be distinguished, the *probability* of the event (which is equally applicable to desirable and undesirable events) and its associated *cost* or *harm* or *incidence* (in the case of disease agents) [46]. Risk assessment requires the quantification of both components through adequate choice of parameters. If a variety of scenarios are available, both these parameters are ideally separately computed to produce risk curves and surfaces [46]. However, in the situation being considered here, a portfolio of scenarios was not available. Consequently, the two parameters were combined in a multiplicative model to calculate the relative expected exposure rate (see below).

Both of these components have several (sub–)components themselves. Most importantly, the probability of a disease cycle establishment event will be determined by at least the ecology of the vector, reservoir, or host species, depending on the type of disease (which may make one or more of these elements irrelevant), and on the probability of occurrence of the parasite. Both these parameters were computed separately and then the results compounded in two different ways.

Risk assessment proceeded in five stages:

1. Ecological risk was computed to quantify probable exposure to the parasite (disease factor) due to the ecological suitability of a cell for disease vectors. This process generated an *ecological relative risk map*.
2. Incidence–based risk was computed to quantify probable exposure to the parasite because of physical contact, that is, due to spatial proximity of one cell to another in which a parasite is known to occur. This process generated an *incidence*–*based relative risk map*.
3. The probability of (human) exposure to a parasite depends on both the ecological risk, which quantifies the probability of vector presence, and the incidence–based risk, which quantifies the likely presence of a parasite. For a more complete risk assessment, even ignoring reservoirs, the effects of these two factors must be jointly analyzed. The first method for this purpose that was used was a multi–criteria analysis in which each factor was taken to be a criterion.
4. The second method of joint analysis was to use a composite risk model that quantitatively combines the ecological and incidence–based risk. This process generated a *composite relative risk map*.
5. The relative expected exposure rate in humans was computed using the composite risk model and the human population in each cell. This process generated a *relative expected exposure rate risk map*.

#### Ecological Risk

For this analysis, ecological risk was quantified by the probability of the presence of a triatomine vector in each cell, that is, 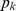 as defined earlier. Since the rest of the analysis had to be performed at the county level, because data at any finer resolution was not available, the average 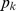 was computed for each of the 254 counties of Texas. In principle, the ecological risk would also incorporate the probability of presence of reservoirs. Such a model of ecological risk has been implicitly [29] and explicitly [30], [31] used to define the minimal ecological conditions required for a disease to spread and establish an autochthonous cycle in a region. If the ecological risk is low, such an establishment is highly unlikely. If that risk is high, then other factors, some of which were modeled below, become critical for establishment.

#### Incidence–Based Risk

It was presumed that incidence–based risk depended on the proximity of a cell to one in which the parasite is present, that is, on spatial correlation. Based on this assumption, incidence–based relative risk was computed using the Besag-York-Mollié (BYM) model [47], [48] which has been widely used for this purpose [37]. This is a Bayesian spatial model which assumes a Poisson sampling distribution for the number of incidences, 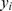 in any area, 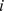. This is appropriate if incidences are rare, as was true in our case. If:
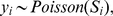
where 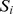 is the relative risk as measured by incidence, the model assumes a Gaussian Markov random field for 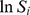 [37], [49]:
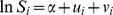
where 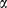 is an average level of relative risk, 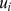 is the correlated heterogeneity, and 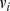 the uncorrelated heterogeneity. Finally, a conditional autoregressive (CAR) model was used for the 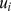 [49]. This model was selected because of its superior performance, as measured by the deviance information criterion (DIC) [50], over a range of data sets in a recent review [37]. The two other models with similar superior performance were more complex semi–parametric models which would have been difficult to parameterize credibly given the lack of more comprehensive data for Chagas disease in Texas.

Model input consisted of an incidence score (0, 1, 2, or 3) for each cell which increased linearly with the number of different types (triatomine vectors, canine hosts, human hosts) in which the parasite was found in a cell (county). Ideally, the exact number of parasites found should be incorporated into the computation but data at that level of detail were not available. Model computations were performed in WinBUGS [51] using code modified from Lawson *et al.* [49]. The CAR model required the specification of a prior, parameterized by the precision, 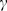, of a multi-variate normal distribution. An uninformative prior (with 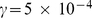) was used because there was no prior information regarding any of the parameters. Model computations were initiated with a “burn–in” of 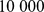 iterations followed by a subsequent 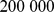 iterations to ensure convergence. Convergence was judged by the lack of autocorrelation after 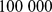 and 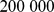 iterations as well as inspection of smooth posterior probability densities for all parameters after 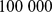 and 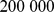 iterations. Model output consisted of a Bayesian posterior probability of relative risk of incidence for each county.

#### Multi–Criteria Analysis

A wide variety of multi–criteria analysis techniques exist [38]; surprisingly, very few have been used in epidemiological contexts. Since the composite risk model discussed next already quantitatively compounds the ecological and incidence–based risk, both interpreted as probabilities, multi–criteria techniques used here were restricted to those that rely entirely on qualitative (*ordinal* or comparative) rankings [52], [53]. Because there was no basis for ordering the two criteria—ecological risk and incidence–based risk—the only method available that is consistent with standard utility theory was *dominance*. One alternative possibility (county, in this case) “dominates” another with respect to risk if it has either higher ecological or incidence–based risk and neither its ecological risk nor its incidence–risk is lower than that of the other alternative (county). The set of non–dominated alternatives is collectively at higher risk than the other alternatives in the sense that none of the other alternatives is worse off than all of the non–dominated alternatives according to every criterion.

However, the technique has well–known problems [52], [54]. All counties that have the highest ecological relative risk or the highest incidence–based relative risk are bound to be non–dominated. To ameliorate this problem, this risk assessment was always used in this analysis along with the results of an analysis that quantitatively compounded these two types of risk. All multi–criteria analysis was done using the MultCSync software package [55].

#### Composite Risk

In contrast to the multi–criteria analysis, the second method of combining ecological risk and incidence–based risk used a multiplicative model to produce a single value of relative risk. Given that what is being computed is the probability component of risk, if both the ecological risk and incidence–based risk are being appropriately interpreted as probabilities (which is reasonable), then, if parasite incidence and vector occurrence are statistically independent, the multiplicative model is appropriate. However, vectors are responsible for introducing the parasite in a cell (even if, as in the case of Chagas disease in Texas, there are other major modes of introduction including migration and transport of contaminated blood [7]). Consequently, quantitative values produced by the multiplicative model must be treated with caution.

#### Relative Expected Exposure Rate

Because no other source of quantitative data was available, we used only one component contributing to the relative expected exposure rate: the potential population that would be exposed to Chagas disease in a county. The populations of the 254 counties were normalized on a scale of 0 to 1 (with 1 being the rank of the county with the highest population). This scaled value was then multiplied by the composite risk which was interpreted as the probability of exposure to the parasite. The result, again normalized to lie between 0 and 1, was interpreted as a relative measure of expected exposure rate. Because of the reservations noted above about the composite risk model's assumption of statistical independence between ecological risk and incidence–based risk, the quantitative estimates produced by this model must be treated with caution. However, it is well–known that the extent to which the housing in an area is built of concrete and similar material (rather than wood, adobe, *etc.*) negatively affects domestic human exposure to triatomines [7], [8]. Spatially georeferenced quantitative data on housing construction in Texas was not available. However, there is some correlation between income levels and housing construction, with higher incomes correlated to concrete housing. Moreover, there is also a correlation between poverty and Chagas disease [7], [14]. Data on median incomes for each county in Texas from the United States was obtained from the Census Bureau [41] and used to refine the results of the expected exposure rate model.

## Results

### Triatomine Biogeography

At the county level, our data collection and collation extended the known distribution of the seven triatomine species in Texas [12] in six cases: *T. gerstaeckeri* to Castro, Galveston, Gonzales, Lubbock, Parker, Victoria, Wilson, and Zapata counties, *T. indictiva* to Hays and Kinney counties, *T. lecticularia* to Bastrop, Blanco, Burleson, Lubbock, and Parker counties, *T. protracta* to Andrews, Bexar, and Terry counties, *T. rubida* to Crane and Upton counties, and *T. sanguisuga* to Bastrop and Kaufman counties. For *T. gerstaeckeri* and *T. lecticularia*, these results extend their ranges to northwest Texas for the first time. Over all, triatomines have now been recorded for 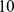 more counties (Andrews, Burleson, Castro, Crane, Galveston, Kaufman, Parker, Terry, Upton, and Wilson) than what was previously established. (Relevant maps are provided in the supplementary materials.)

### Species Distribution Models

Model performance was judged using the test AUC, that is, the area under the receiver operating characteristic (ROC) curve and a set of internal binomial tests in the Maxent software package [26]. All three species produced test AUC values above the threshold of 0.9: averaged over the 100 replicate models, 0.979 for *T. gerstaeckeri*, 0.924 for *T. sanguisuga*, and 0.959 for *T. lecticularia*. On the average, all binomial tests were significant (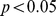). Because the models for *T. lecticularia* were constructed using only 11 presence records, the fact that its average AUC, besides being high, was greater than that of *T. sanguisuga*, suggests that model predictions are reliable. Moreover, a recent study indicates that models constructed using the Maxent algorithm are reliable so long as there are more than 10 presence records [56].

**Figure 2:**
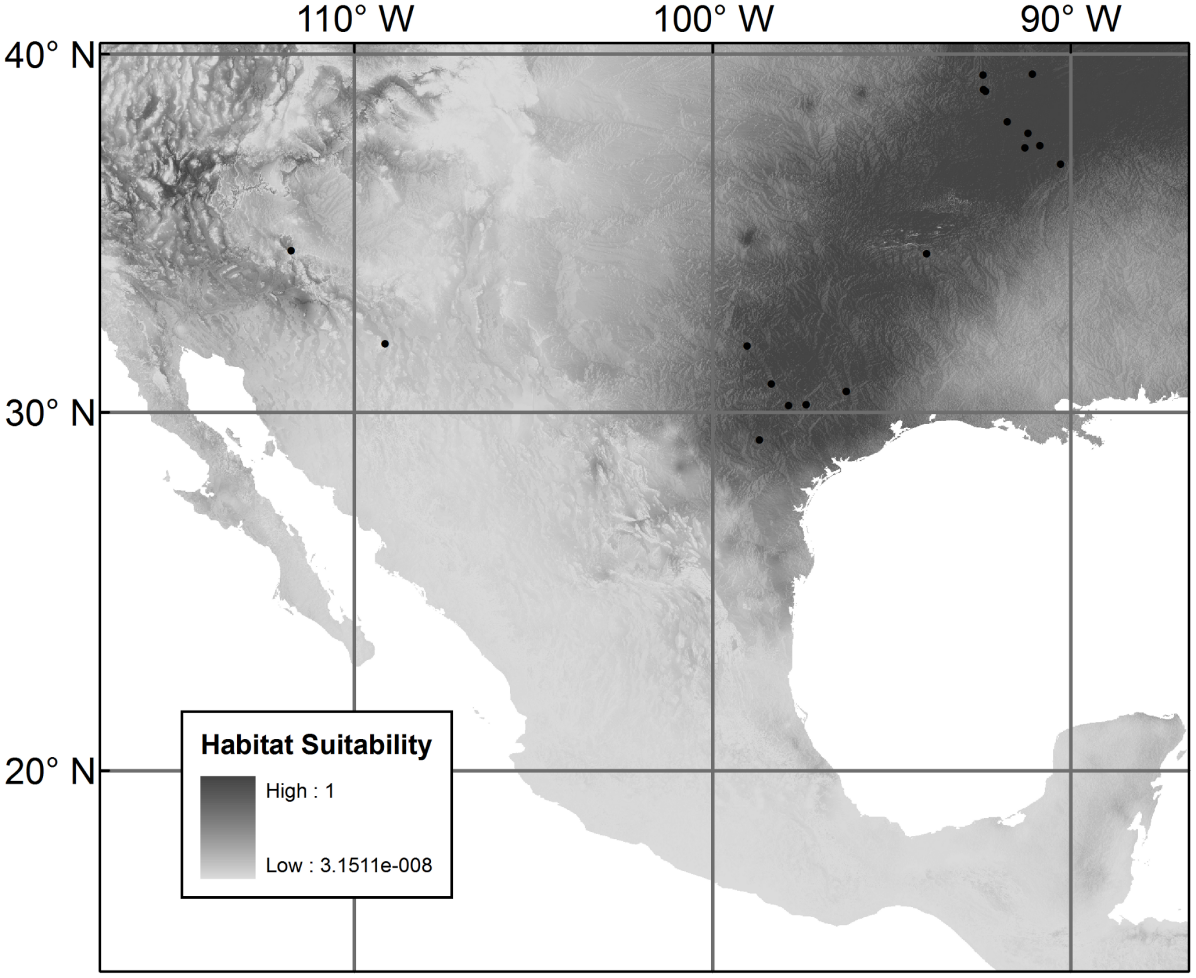
Species distribution model for *Triatoma sanguisuga*. The black dots show the occurrence points used for model construction. Much of the distribution is predicted to be in the eastern United States where the species has been collected from Texas to Florida, However, because of the two occurrence points to the west, a disjoint western distribution is also predicted and merits further investigation, as noted in the text.

**Figure 3:**
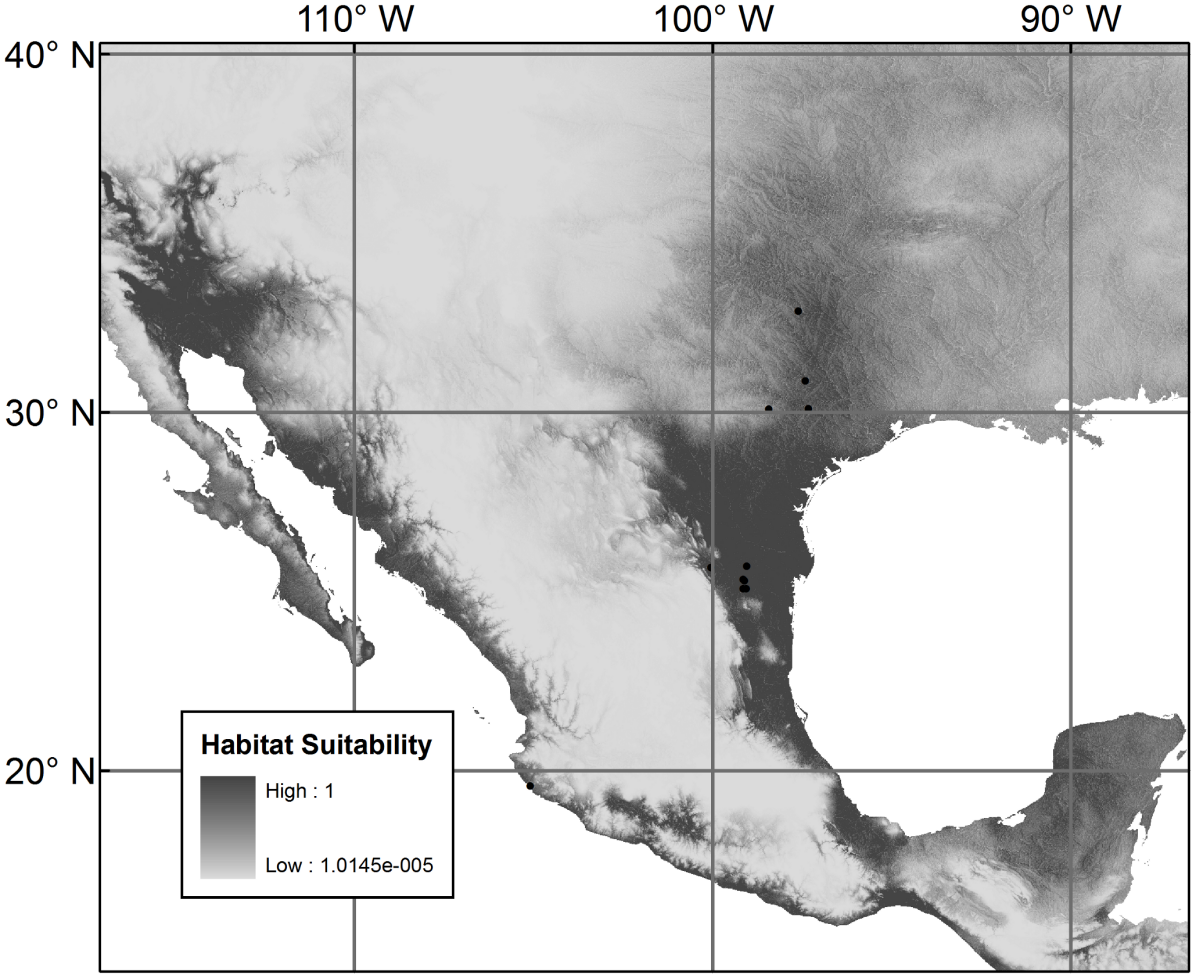
Species distribution model for *Triatoma lecticularia*. The black dots show the occurrence points used for model construction. The distribution is diffusely spread along both the east and west coasts.

Figures 1, 2, and 3 show the three species distribution models, respectively. For *T. gerstaeckeri*, four out of 74 occurrence records fell in cells with habitat suitability 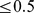, for the other species, there was in each case one such record. The presence of a limited number of anomalous points is expected because species are often found in sub-optimal habitats, especially at the geographical margins of their ranges [54], [57], as was the case with our points. The model for *T. gerstaeckeri* conforms with what is known about the distribution of the species from field records though it differs from the older model of Beard *et al.* [16] (see Discussion). There is a high probability of occurrence 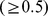 in the southern United States, especially in and around Texas, as well as in northeast México. For *T. sanguisuga*, the two occurrence points from the west (obtained from museum collections) have the effect of predicting suitable habitat in the western United States and México where the species has been collected in Arizona, California, and México [8], [39], [58]. *T. lecticularia* has a widespread predicted distribution along both coasts of North America but remains rare in collections along the western coast where all of our records came from México. Lent and Wygodzinsky [39] included New Mexico in the distribution of *T. lecticularia* but the provenance of those data remains unknown. There appears to be no recent record of the species in New Mexico and predicted highest habitat suitability is only 0.16.

### Probabilistic Risk Analysis

**Figure 4:**
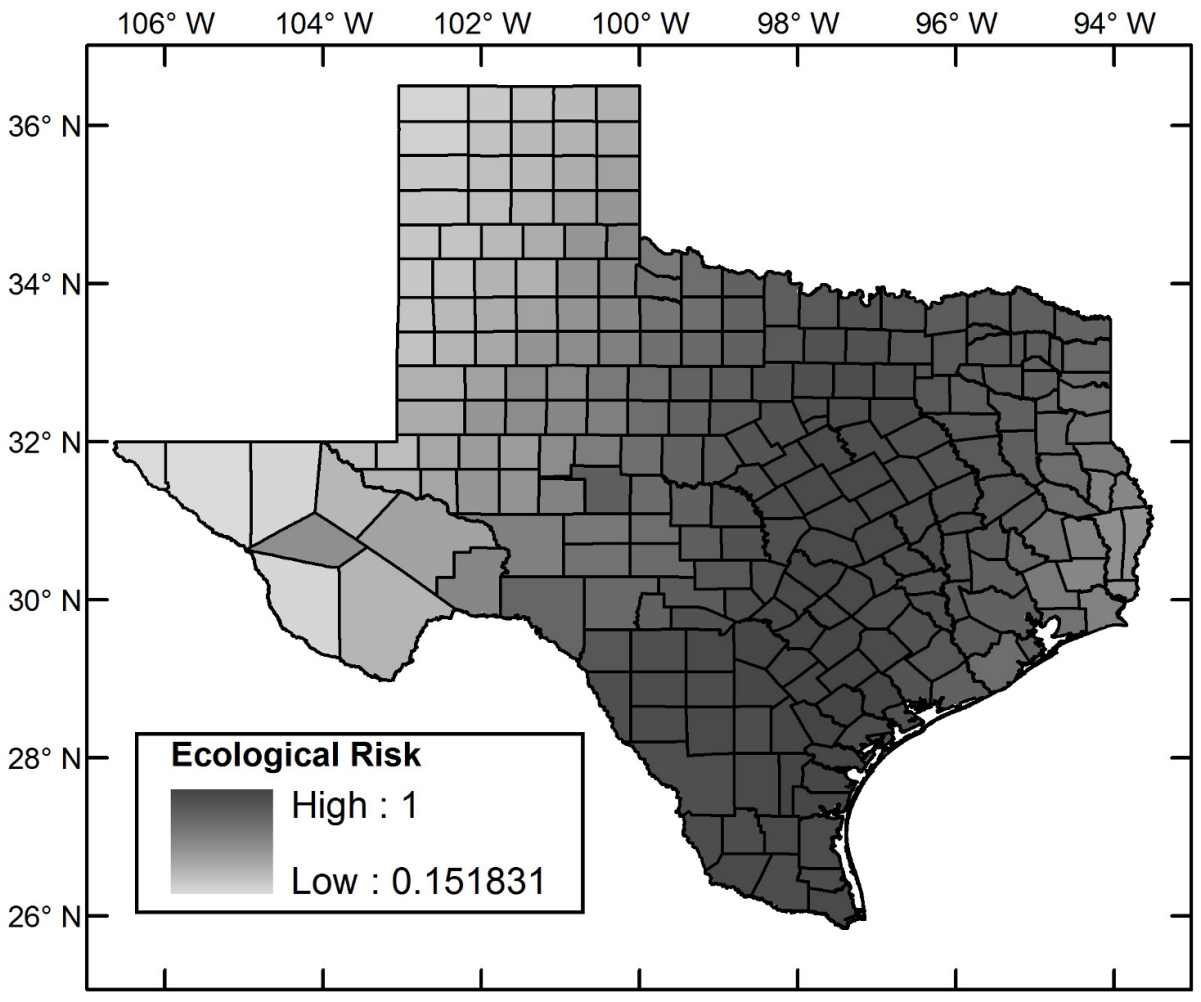
Ecological risk map for Chagas Disease in Texas.

**Figure 5:**
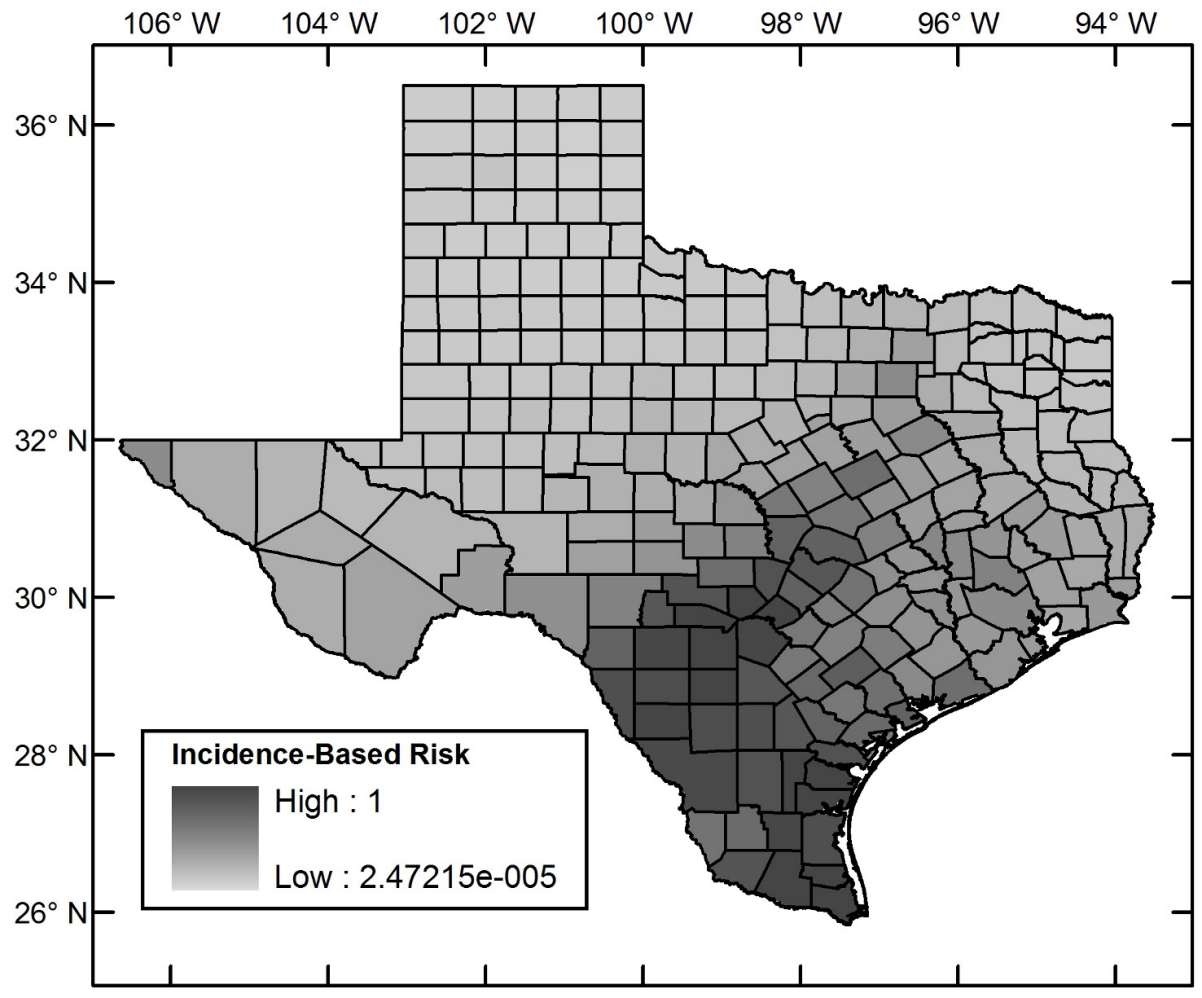
Incidence–based risk map for Chagas Disease in Texas.

**Figure 6:**
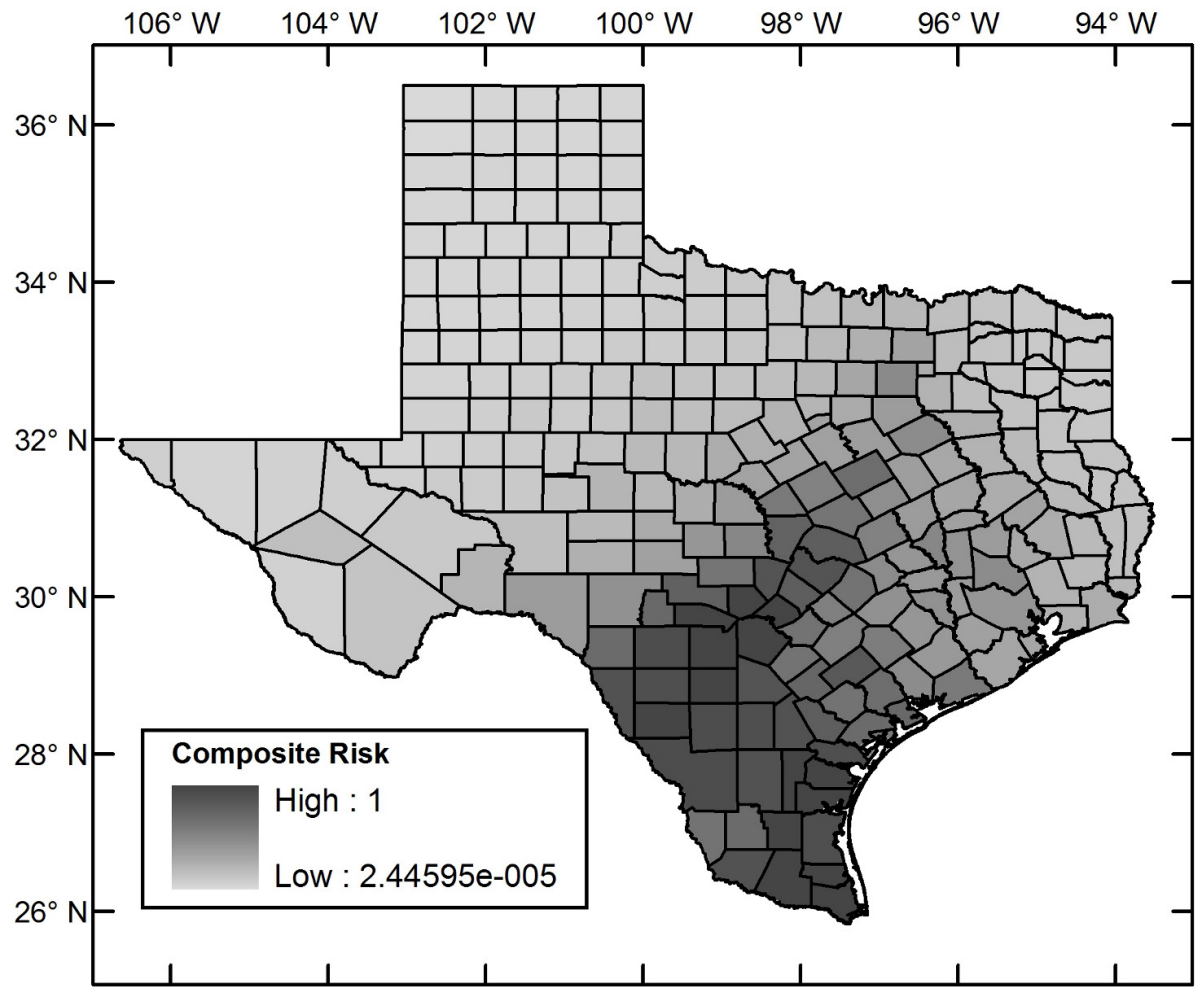
Composite risk map for Chagas Disease in Texas.

Figure 4 shows the (relative) ecological risk map for the region including Texas. Figure 5 shows the incidence–based risk map for Texas, and Figure 6 the composite risk map. Table 2 shows the counties with the highest risk in each of these categories. Compared to the incidence-based risk map, the composite risk map lowers the relative risk of counties to the far west and north of Texas because, even though *T. cruzi* has been reported in these areas, the habitat suitability for the triatomines remains low.

**Table 2:** High Chagas risk counties in Texas.

Ecological	Incidence–Based	Composite	Expected Exposure
Jim Wells	Cameron	Cameron	Bexar
Bee	Nueces	Nueces	Harris
Goliad	Kleberg	Kleberg	Hidalgo
De Witt	Hidalgo	Hidalgo	Dallas
Nueces	Jim Wells	Jim Wells	Travis
Wilson	Willacy	Wiilacy	Cameron
San Patricio	Dimmit	Medina	Nueces
Live Oak	Medina	Dimmit	Tarrant
Karnes	Bandera	Frio	Williamson
Guadalupe	Frio	Bandera	Collin

When we consider ecological risk and incidence–based risk separately in the multi–criteria dominance analysis, instead of compounding them to compute the composite risk, three counties are in the non–dominated set: Cameron, Jim Wells, and Nueces. All of these counties have incidences of *T. cruzi*. When this analysis is restricted to counties with no report as yet of *T. cruzi*, the non-dominated set consists of Goliad, Kenedy, and Wilson counties. This means that these three counties have high suitability for the presence of vector species as well as spatial contiguity to *T. cruzi* occurrences and are foci of special concern for Chagas disease.

When we consider together both non–dominated sets and the top five counties according to the ecological, incidence–based, and composite risk maps, eleven counties are selected (Bee, Bexar, Brooks, Cameron, DeWitt, Goliad, Hidalgo, Jim Wells, Kenedy, Kleberg, and Nueces) and all are in south Texas in an almost contiguous cluster starting at the Mexican border. When we include the top ten counties, an additional nine counties (Bandera, Dimmit, Frio, Guadalupe, Karnes, Live Oak, Medina, San Patricio, and Willacy) are selected; once again, all of these counties are from south Texas.

### Relative Expected Exposure Rate Risk Map

**Figure 7:**
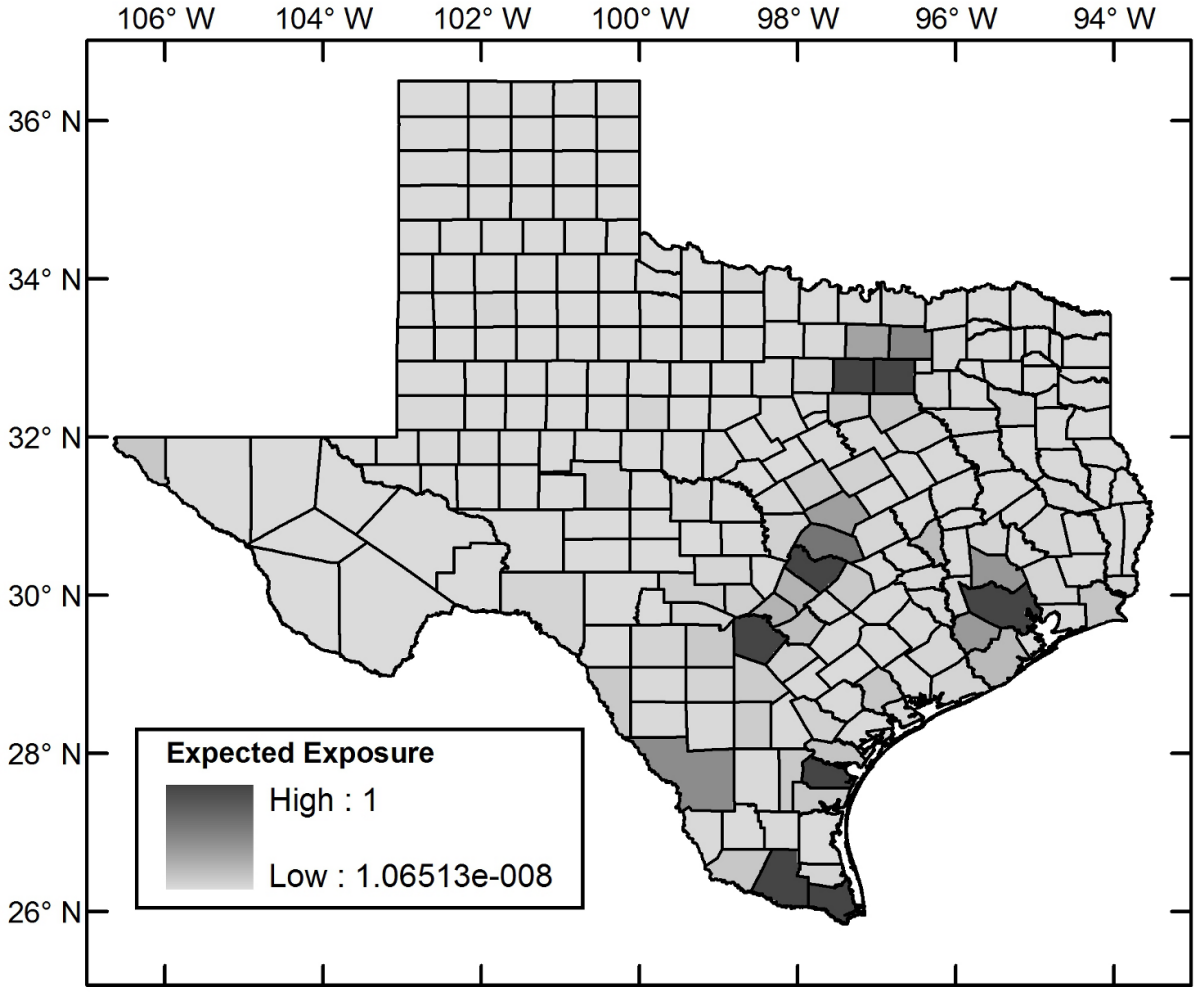
Expected exposure risk map for Chagas Disease in Texas.

Figure 7 shows the relative expected exposure rate at the county level. If the top five counties are added to the list of high risk counties, three counties outside south Texas are added: Dallas (north Texas), Harris (east Texas), and Travis (central Texas), because of the high human populations. If ten such counties are used, three additional counties outside south Texas are included (Collin and Tarrant in north Texas and Williamson in central Texas). Thus, consideration of human population density in a multiplicative model leads to a slightly more widespread attribution of risk than ecological and incidence–based risk. Nevertheless, the focus on south Texas remains strong. Moreover, only two of the high risk counties were ranked very low by median income using 2006 data from the United States Census Bureau [41]—Cameron and Hidalgo, which ranked 228 and 234, respectively, out of 254 counties. Both of these are in south Texas. Low median income is likely to be indicative of relatively poorer living conditions and possible lack of concrete housing. Thus housing and living conditions, which were not quantitatively modeled, also implicate south Texas as the area of highest risk.

## Discussion

For *T. gerstaeckeri*, our model predicted much more highly suitable habitat (high probability of occurrence) in central and east Texas and less in northwest Texas than the earlier model of Beard *et al.* [16] and is more consistent with the distribution map created by Kjos *et al.* [12] on the basis of collection records, including our extension of that distribution map with additional occurrence records (see Figure S1). The better performance of our model is presumably due to the availability of many more occurrence records from the United States for this species. Moreover, our model also predicted more suitable habitat for this species in México than the earlier model. This suggests an enhanced focus on this species for the control of Chagas disease in both Texas and north México.

Data collection projects are in place for all triatomine species in the southern United States and in México over the next five years. (See Figures S2–S6 for new occurrence records for *T. indictiva*, *T. lecticularia*, *T. protracta*, *T. rubida*, and *T. sanguisuga*, respectively.) All model predictions will be tested in the field, in particular, the limits of the western distributions of *T. lecticularia* and *T. sanguisuga*. Part of the importance of model construction is to provide testable hypotheses that guide survey design, and the results reported here will be used for that purpose.

All risk maps point to one unsurprising but nevertheless important conclusion: to the extent that there is risk for Chagas disease in the United States, one important focus is south Texas. Given the relative absence of reported autochthonous disease cases elsewhere (only three such cases have been confirmed outside Texas), it is the most important region of concern.

The methods used in this analysis do not provide a quantitative estimate of absolute risk or expected exposure rate, which is typically hard to produce in any context and the problem is amplified for diseases on which information is not being systematically collected. What it does provide is the relative risk in one unit compared to other spatial units at the county level. Nevertheless, the critical review of Bern and Montgomery [11] of all available data on Chagas disease in the United States strongly suggests that the absolute risk is also high.

The first three recommendations made below are geared towards obtaining the kind of data that would permit quantitative absolute risk assessment. However, the fourth recommendation, requiring the testing of blood donations, presumes that the absolute risk is high, and this needs some justification. Blood transfusion has been etiologically important as a source of Chagas disease along with immigration from areas of high Chagas disease incidence and an autochthonous cycle [11]. Currently, the American Association of Blood Banks (AABB) recommends such tests but does not require them. Testing began in 2007 using a test licensed by the United States Federal Drug Administration, in December 2006. Major laboratories that account for more than 65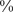 of the total blood collected in the United States already carry out such tests (http://www.aabb.org/Content/Programs_and_Services/Data_Center/Chagas; last accessed 28 February 2010). The fourth recommendation is to extend coverage to the remaining 35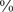 for the high risk areas of Texas. There are two arguments against mandatory testing: (i) the added cost; and (ii) the potential for false positive units to be removed from the blood supply. These costs must be compared to the benefits of testing. A simulation model developed by the Office of Biostatistics and Epidemiology, Center for Biologics Evaluation and Evaluation, United States Food and Drug Administration in 2009 predicted that, with no testing, there would be about 44 cases of transmission–induced Chagas disease in the United States each year (Richard Forshee, personal communication; www.fda.gov/downloads/AdvisoryCommittees/CommitteesMeetingMaterials/BloodVaccinesandOther-Biologics/BloodProductsAdvisoryCommittee/UCM155628.pdf). With 65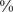 testing, that reduces to about 15 cases. These numbers are sufficiently high to suggest that areas with high relative risk, which would contribute disproportionately more cases, should have mandatory testing. Moreover, if testing is restricted to only high relative risk areas, rather than the entire blood supply, the cost and the potential loss of false positive test units are lower. Unfortunately, data to quantify these arguments are presently not available.

### Recommendations

On the basis of this analysis, we make the following five recommendations:

- Given the risk assessment of this paper, it is imperative that Chagas disease be designated as reportable in Texas as it has been in Arizona since 2007 and Massachusetts since 2008. Additional systematic data acquisition on disease cases will permit more complete risk assessments, including those of absolute risk, in the future which, in turn, can guide the formulation of optimal public health initiatives to prevent disease. This is the most important recommendation from this analysis.
- For the same reasons, the serological status of human and canine populations should be investigated in south Texas, especially in counties south of 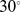
N of latitude, because of the enhanced relative risk for Chagas disease in that region. This is particularly relevant for the high risk counties identified by the multi–criteria analysis in which *T. cruzi* incidence is yet to be detected: Goliad, Kenedy, and Wilson, since it is highly likely that the parasite is present but unrecorded. Such an investigation has a dual purpose: (i) to prevent the occurrence of Chagas disease and related complications in positive individuals to the extent that it is possible, given the paucity of medical interventions available [7]; and (ii) to help formulate preventive strategies for the establishment (beyond current endemicity) and spread of Chagas disease. Humans and canines must both be monitored because of their competence as reservoirs for *T. cruzi* [7]. In high risk areas, as identified by multi–criteria analysis and by the composite risk model, vector species are very likely present in sufficient numbers; consequently, it is important to monitor reservoir species.
- Similarly, in order to prevent establishment and spread of Chagas disease, wild reservoir species, especially rodents (such as Neotoma species, which are confirmed highly competent reservoirs) merit investigation and monitoring in high risk areas.
- The testing of blood donors for antibodies to Chagas disease should be made mandatory at least in high risk areas (once again, at least in the counties south of 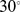
N of latitude in Texas). The reasons for making this recommendation were explained earlier.
- The Southern Cone Initiative [4], [6] has interrupted the transmission of Chagas disease in several South American countries; similar efforts are being attempted for the countries of central America [6], [7]. We recommend that a similar international initiative be undertaken by the United States and México in the trans–border region (and not restricted to Texas within the United States) to control the spread of Chagas disease. Ideally, such an initiative should not be restricted to only Chagas disease. Rather, it should include the entire spectrum of vector–borne diseases capable of spreading across the border including dengue, lesihmaniasis, tick–borne diseases, and West Nile virus, besides Chagas, to develop, for instance, integrated strategies of arthropod vector and, in some cases, reservoir control.

### Limitations

Finally, beyond those discussed in the Materials and Methods section, eight other limitations of this analysis should be explicitly noted:

1. This analysis only partly incorporated risk from non–autochthonous Chagas disease occurrence and transmission in Texas. (It used data on confirmed parasite incidences of any provenance.) Important mechanisms include blood transfusion and immigration from areas of high Chagas disease incidence [10]. At present it remains impossible to provide reliable quantitative estimates of these risks. What remains important, however, even if the disease is introduced through these mechanisms, its potential to establish itself through an autochthonous cycle will depend on the probability computations reported here, in particular, the ecological risk model.
2. This analysis did not consider all vector species for Chagas disease in Texas. Moreover, it is possible that beyond these seven species that are known to occur in Texas (and besides *T. recurva*), other species found in northern México may also be present in Texas according to recent models [27]. However, given the absence or rarity of these species in the museum collections we investigated, it does not appear very likely that this possibility will be realized. Further, global factors such as climate change may result in species' range shifts. Assessing risk from this possibility was beyond the scope of this paper.
3. This analysis assumed that *T. gerstaeckeri*, *T. lecticularia*, and *T. sanguisuiga* are equally competent as vectors of T. cruzi. While there is no evidence against such an equivalence, it has not been established through experiment. The assumption was made here in the absence of any alternative.
4. This analysis did not consider well–known differences between *T. cruzi* strains/ types in the etiology of Chagas disease [7], [13]. Not enough information was available on the spatial distribution of the different strains anywhere in the study area to assess the significance of these differences.
5. It was assumed here that competent reservoirs for Chagas disease were always present and differences of reservoir occurrence between cells can be ignored. The reason for this assumption was the establishment of Chagas disease in domestic and peridomestic cycles and the abundance of wild reservoirs in non–urban regions of Texas. However, a spatially variable large parasite burden in wild reservoir species would require a revision of the relative risk estimates reported here. At present there is no evidence for the existence of such a factor.
6. The analysis did not consider vector population dynamics which have been shown to be important in the disease cycle [59]. Unlike many regions of South America, there was no information available for this study area to introduce these complications. Moreover, at the level of spatial resolution of this analysis the effects of population dynamics are probably not as important as habitat suitablity of the vector species of Chagas disease.
7. The BYM model only takes spatial contiguity into account in the computation of spatial risk. It does not take quantitative spatial information (for instance, distances between points) into account. Unfortunately enough data did not exist to attempt a more sophisticated spatial analysis of risk.
8. Species distribution models only predict species' probable presence or absence, and do not predict abundance. This is an important limitation because epidemiological models typically predict that disease establishment is likely to depend on the abundance of vector (as well as reservoir, host, *etc.*) species. An implicit assumption of the modeling techniques used here is that, beyond a straightforward relationship with probability occurrence, habitat suitability (as predicted by species distribution models) is also correlated with a species' abundance. However, field studies to test this assumption are yet to be reported. For the Yucatán peninsula of México, there have been previous risk assessments based on modeled vector species abundance [60] (using *T. dimidiata*, the most important vector in Mesoamerica [7]) but the required data were not available for this study area.

Finally, one methodological innovation of this analysis should be noted since it is likely to be relevant to other contexts. This is the use of multi–criteria dominance analysis to identify high risk areas. In general, formal decision analysis has been surprisingly sparingly used in epidemiological contexts. However, techniques developed in that field can provide comprehensive decision support whenever complex decisions have to be analyzed. Here, we used one of the simpler multi–criteria techniques, the computation of non–dominated alternatives, to identify counties which are at high risk from Chagas disease even though the parasite has not yet been reported from them. Other, model–based techniques, selected the same region as areas of concern in south Texas. When used together to produce identical or similar results, these strategies lead to a more robust estimation of relative risk than otherwise possible. The strategy is fully general and can be exported to other contexts in which computing and mapping disease relative risk is of interest.

## Supporting Information

Figure S1 New counties for *Triatoma gerstaeckeri*. The new counties are shown in dark gray and labeled by name.
(0.48 MB TIF)

Figure S2 New counties for *Triatoma indictiva*. The new counties are shown in dark gray and labeled by name.
(0.32 MB TIF)

Figure S3 New counties for *Triatoma lecticularia*. The new counties are shown in dark gray and labeled by name.
(0.40 MB TIF)

Figure S4 New counties for *Triatoma protracta*. The new counties are shown in dark gray and labeled by name.
(0.39 MB TIF)

Figure S5 New counties for *Triatoma rubida*. The new counties are shown in dark gray and labeled by name.
(0.34 MB TIF)

Figure S6 New counties for *Triatoma rubida*. The new counties are shown in dark gray and labeled by name.
(0.43 MB TIF)

Table S1 *Trypanosoma cruzi* incidence in Texas by county.
(0.07 MB PDF)

Table S2 Species records in the Disease Vectors Database.
(0.05 MB PDF)
