# Supplementary figures and images for: Chagas Disease Risk in Texas
Source: PLoS Negl Trop Dis. 2010 Oct 5;4(10):e836. doi: 10.1371/journal.pntd.0000836 (PMC2950149; doi:10.1371/journal.pntd.0000836)

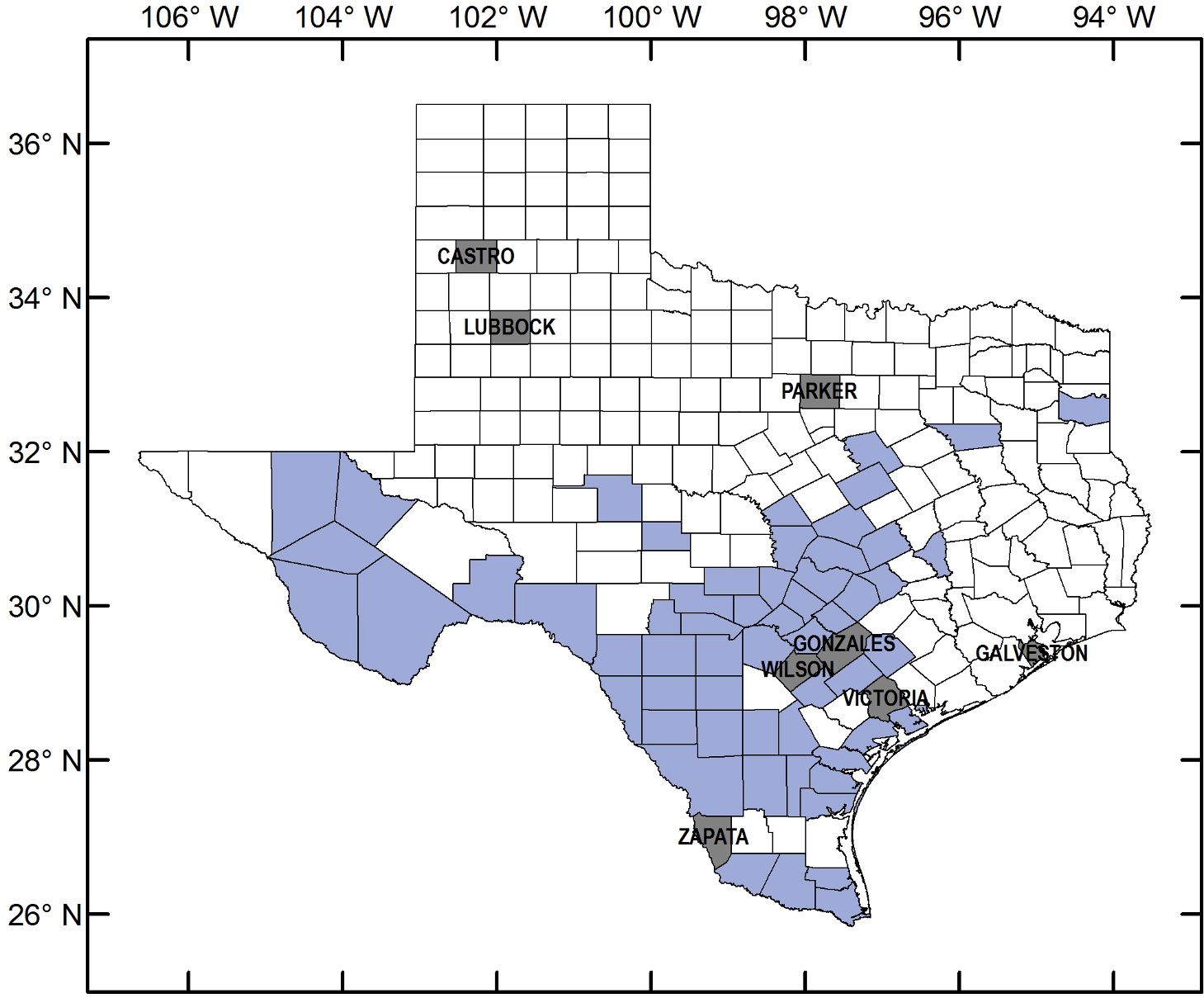

Supplement: Figure S1 — New counties for Triatoma gerstaeckeri. The new counties are shown in dark gray and labeled by name. (0.48 MB TIF) [file pntd.0000836.s001.tif]

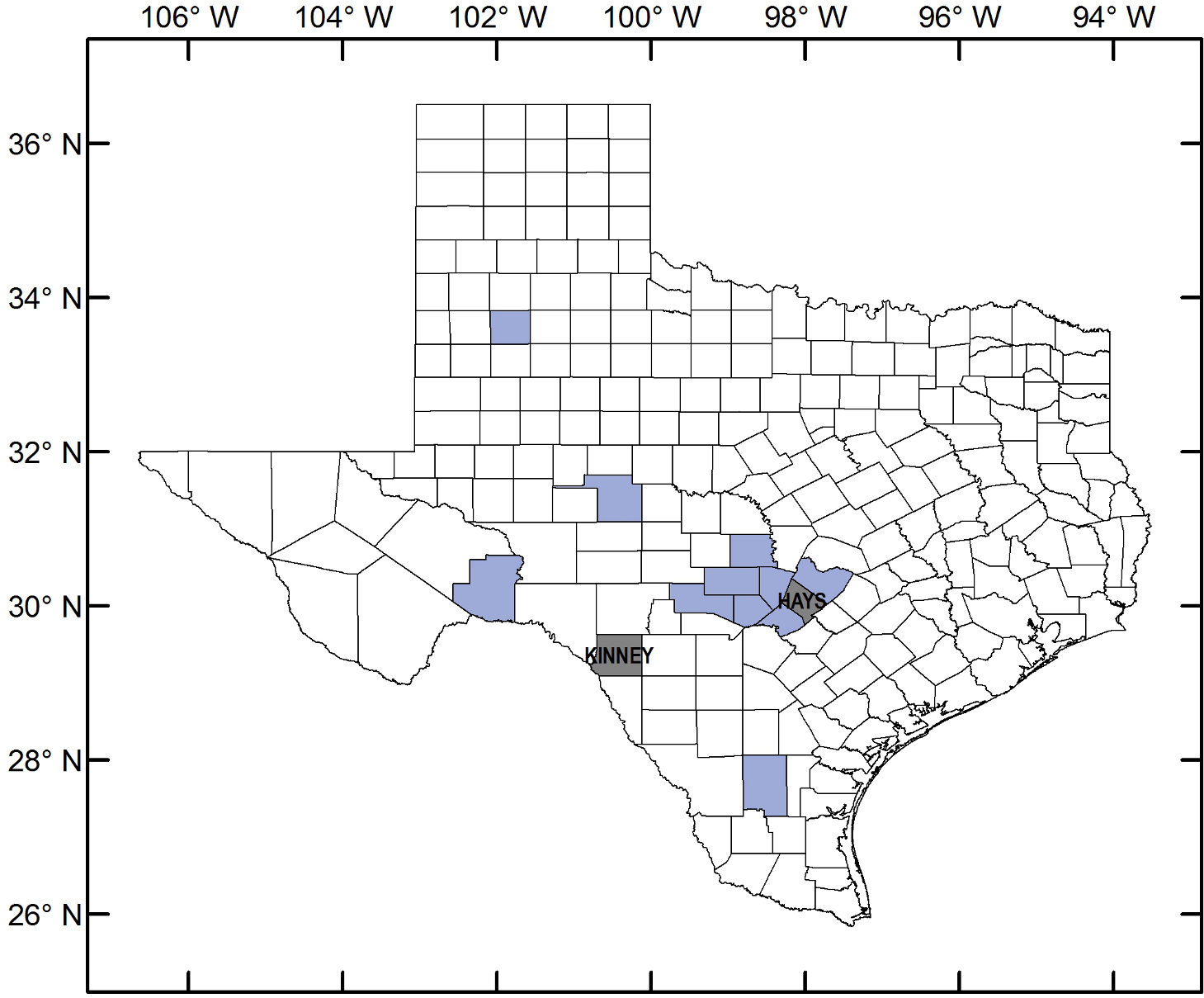

Supplement: Figure S2 — New counties for Triatoma indictiva. The new counties are shown in dark gray and labeled by name. (0.32 MB TIF) [file pntd.0000836.s002.tif]

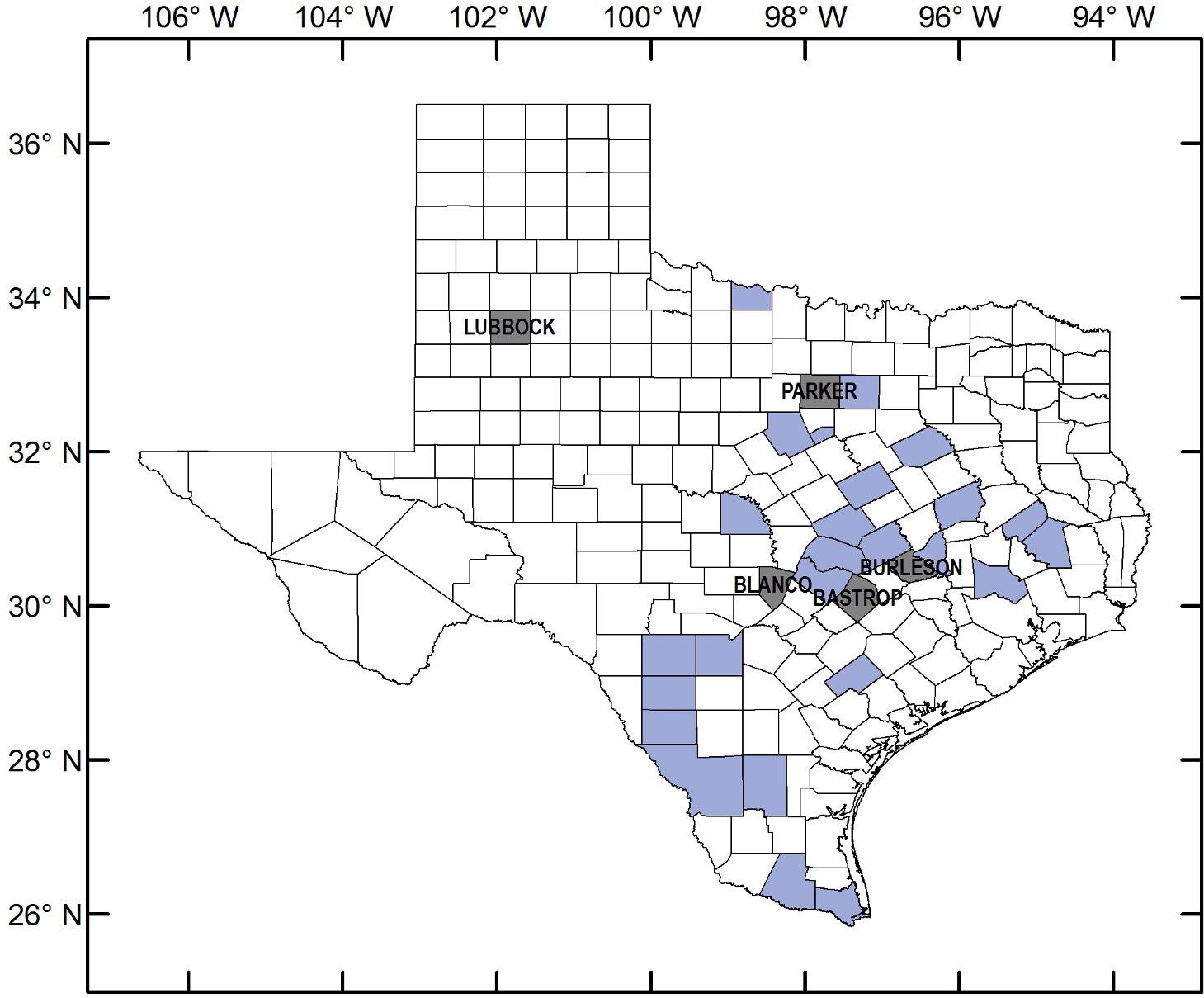

Supplement: Figure S3 — New counties for Triatoma lecticularia. The new counties are shown in dark gray and labeled by name. (0.40 MB TIF) [file pntd.0000836.s003.tif]

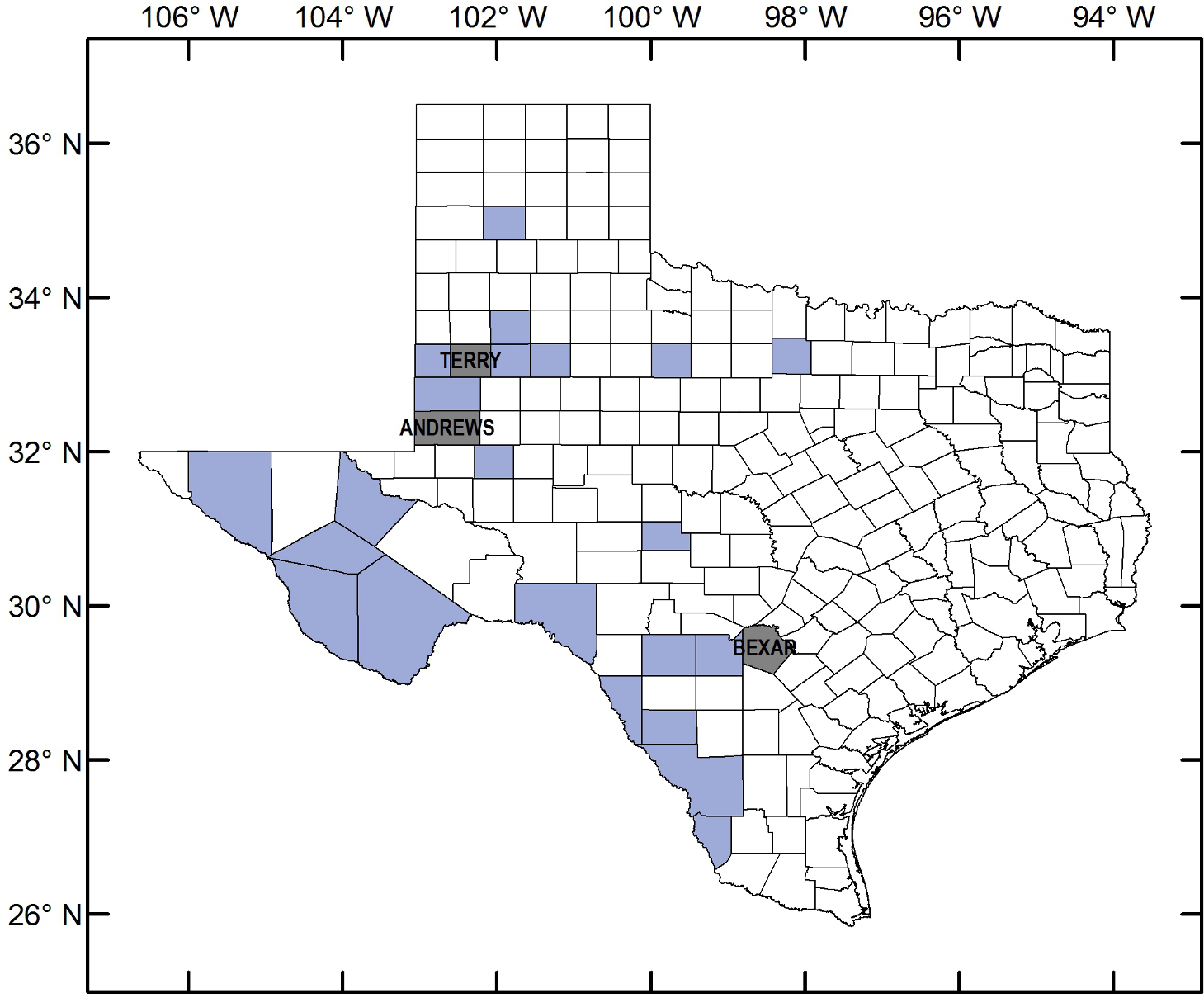

Supplement: Figure S4 — New counties for Triatoma protracta. The new counties are shown in dark gray and labeled by name. (0.39 MB TIF) [file pntd.0000836.s004.tif]

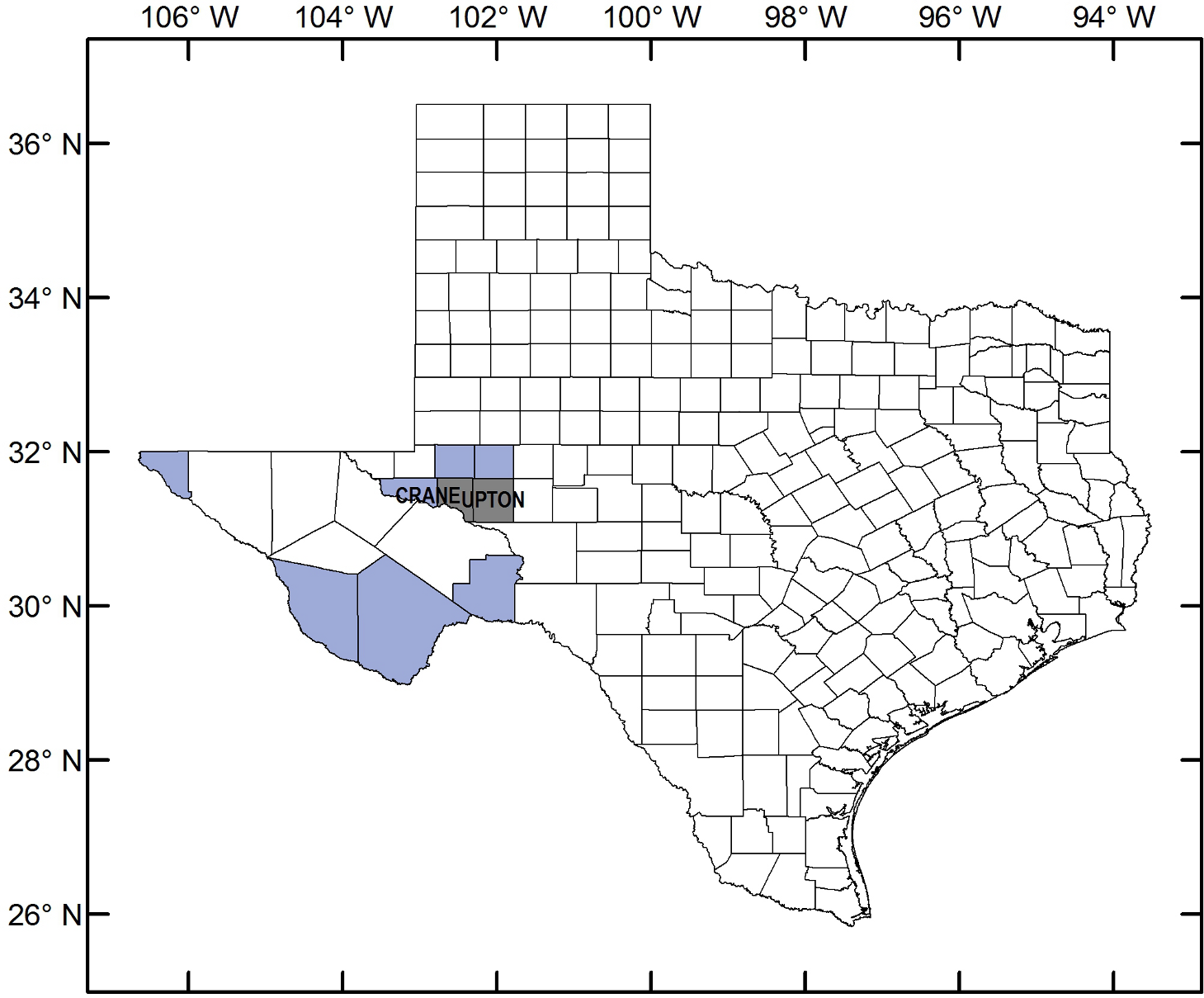

Supplement: Figure S5 — New counties for Triatoma rubida. The new counties are shown in dark gray and labeled by name. (0.34 MB TIF) [file pntd.0000836.s005.tif]

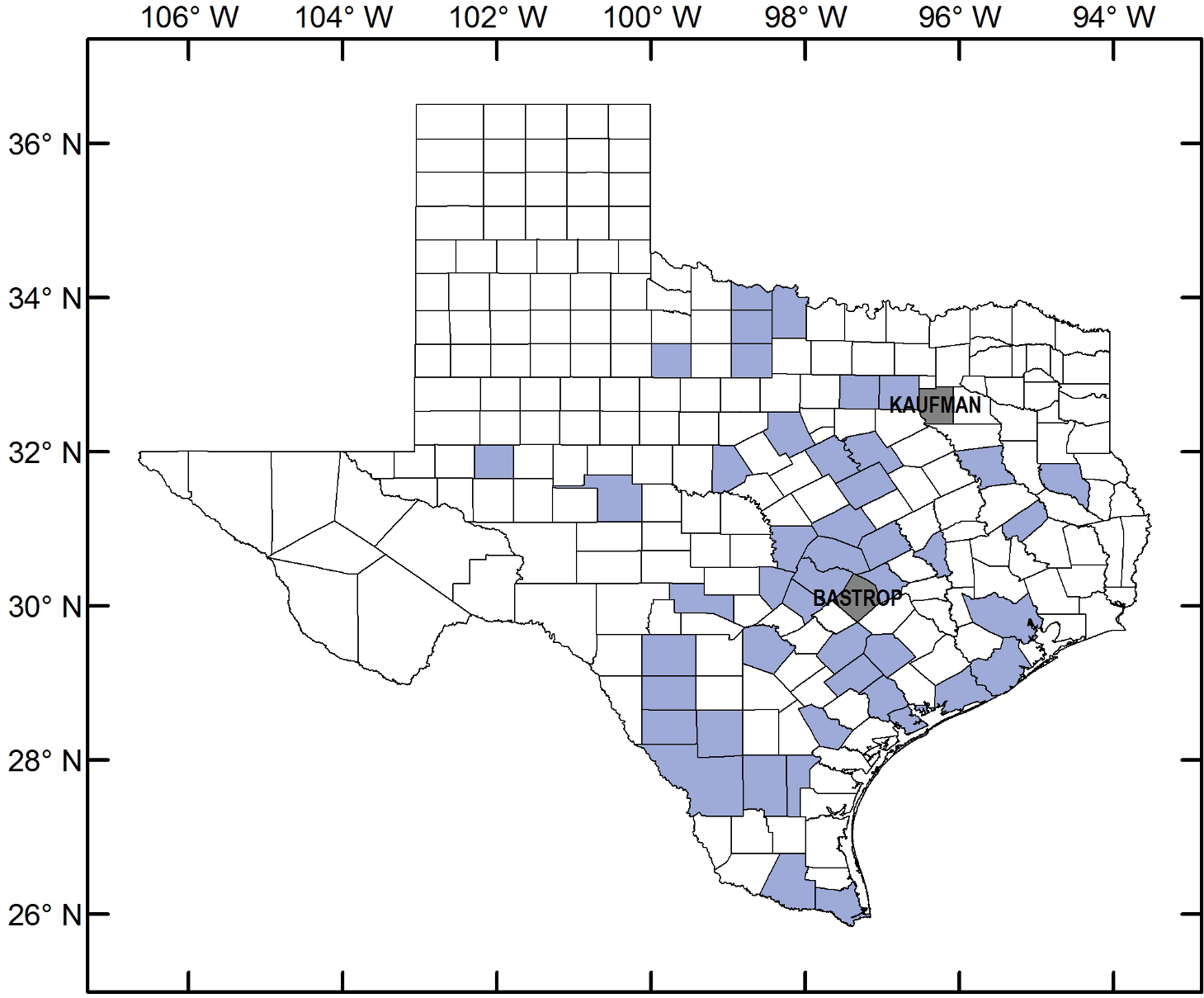

Supplement: Figure S6 — New counties for Triatoma rubida. The new counties are shown in dark gray and labeled by name. (0.43 MB TIF) [file pntd.0000836.s006.tif]
