# Supplementary material for: Chagas Disease Risk in Texas
Source: PLoS Negl Trop Dis. 2010 Oct 5;4(10):e836. doi: 10.1371/journal.pntd.0000836 (PMC2950149; doi:10.1371/journal.pntd.0000836)
Supplement: Table S1 — Trypanosoma cruzi incidence in Texas by county. (0.07 MB PDF) [file pntd.0000836.s007.pdf]

Chagas Disease Risk in Texas

Sahotra Sarkar[1,2,*], Stavana E. Strutz[1], David M. Frank[2], Chissa–Louise Rivaldi[1], Blake Sissel[1], Victor Sánchez–Cordero[3]
1 Section of Integrative Biology, University of Texas, Austin, TX 78712, USA
2 Department of Philosophy, University of Texas, Austin, TX 78712, USA
3 Instituto de Biología, Universidad Nacional Autónoma de México, México City, México 04510
∗ Section of Integrative Biology, University of Texas, Austin, TX 78712, USA; Phone: 1 512 232 3800; FAX: 1 512 471 4806; E-mail: sarkar@mail.utexas.edu

Table S1

***Trypanosoma cruzi* Incidence in Texas by county**. Note that our extensive literature search generated 82 counties instead of the 64 reported by Hanford *et al.* [1]. As in the rest of this paper, only post–1960 data are included.

County	No. of Records	Host Species	Reference
Aransas	3	Canine	[2]
Atascosa	8	Canine	[2]
Austin	2	Canine	[2]
Bandera	≥ 1	Triatoma	[3]
	3	Canine	[2]
Bastrop	≥ 1	Triatoma	[3]
Bee	17	Canine	[2]
Bell	≥ 1	Triatoma	[3]
Bexar	≥ 1	Triatoma	[3]
	544	Triatoma	[4]
	65	Canine	[2]
	1	Human	[5, 6]
Blanco	≥ 1	Triatoma	[3]
	3	Canine	[2]
Brazos	≥ 1	Triatoma	[3]
	48	Canine	[2]
Brewster	≥ 1	Triatoma	[3]
Brooks	3	Canine	[2]
Brown	1	Canine	[2]
Burnet	≥ 1	Triatoma	[3]
	4	Canine	[3]
Calhoun	≥ 1	Triatoma	[3]
Cameron	≥ 1	Triatoma	[3]
	24	Triatoma	[7]
	161	Triatoma	[8]
	41	Canine	[2]
	1	Human	[1, 9]
Collin	≥ 1	Triatoma	[1]
	1	Canine	[10]
Comal	≥ 1	Triatoma	[3]
	9	Canine	[2]

Coryell	1	Canine	[2]
Dallas	≥ 1	Triatoma	[1]
	5	Canine	[2]
	1	Human	[11]
DeWitt	1	Canine	[3]
	2	Canine	[2]
Dimmit	≥ 1	Triatoma	[3]
	3	Canine	[2]
Duval	≥ 1	Triatoma	[3]
Ellis	≥ 1	Triatoma	[1]
El Paso	≥ 1	Triatoma	[3]
Frio	≥ 1	Triatoma	[3]
	4	Canine	[2]
Gillespie	≥ 1	Triatoma	[3]
Grimes	3	Canine	[2]
Harris	20	Canine	[2]
	1	Human	[12]
	1	Human	[13]
	22	Human	[14]
	6	Human	[15]
Hays	4	Canine	[2]
Henderson	1	Canine	[2]
Hidalgo	≥ 1	Triatoma	[3]
	11	Canine	[2]
	1	Human	[9]
Howard	≥ 1	Triatoma	[1]
Jasper	≥ 1	Triatoma	[1]
Jeff Davis	1	Canine	[2]
Jefferson	1	Canine	[2]
Jim Wells	67	Canine	[2]
	1	Human	[1]
Jones	≥ 1	Triatoma	[1]
Karnes	24	Canine	[2]
Kendall	≥ 1	Triatoma	[3]
	4	Canine	[2]
Kerr	≥ 1	Triatoma	[3]
	1	Canine	[2]
Kinney	≥ 1	Triatoma	[3]
Kleberg	≥ 1	Triatoma	[3]
	19	Canine	[2]
	1	Human	[1]
Lampasas	≥ 1	Triatoma	[3]
	1	Canine	[2]
La Salle	≥ 1	Triatoma	[3]
Lavaca	≥ 1	Triatoma	[3]
Live Oak	≥ 1	Triatoma	[1]
Lubbock	1	Canine	[2]
McLennan	≥ 1	Triatoma	[3]
	3	Canine	[2]

	3	Human	[1, 16]
McMullen	≥ 1	Triatoma	[1]
Mason	≥ 1	Triatoma	[1]
Matagorda	≥ 1	Triatoma	[3]
	2	Canine	[2]
Maverick	≥ 1	Triatoma	[3]
Medina	≥ 1	Triatoma	[3]
	4	Canine	[2]
Menard	≥ 1	Triatoma	[3]
Milam	≥ 1	Triatoma	[3]
Montgomery	≥ 1	Triatoma	[3]
	6	Canine	[2]
Navarro	≥ 1	Triatoma	[3]
	6	Triatoma	[17]
	≥ 1	Canine	[17]
Nueces	≥ 1	Triatoma	[3]
	4	Triatoma	[18]
	22	Canine	[2]
	1	Human	[1]
	3	Human	[19]
	9	Human	[18]
Orange	≥ 1	Triatoma	[1]
Polk	≥ 1	Triatoma	[1]
Potter	≥ 1	Triatoma	[1]
	1	Human	[1]
Real	≥ 1	Triatoma	[3]
Refugio	≥ 1	Triatoma	[3]
Runnels	1	Canine	[2]
San Patricio	5	Canine	[2]
	1	Human	[1, 20]
Somervell	1	Canine	[2]
Starr	≥ 1	Triatoma	[3]
Tarrant	1	Canine	[2]
Taylor	≥ 1	Triatoma	[1]
Terrell	≥ 1	Triatoma	[3]
Tom Green	≥ 1	Triatoma	[3]
Travis	≥ 1	Triatoma	[3]
	38	Canine	[2]
Trinity	≥ 1	Triatoma	[3]
Uvalde	≥ 1	Triatoma	[3]
	29	Canine	[2]
Val Verde	≥ 1	Triatoma	[3]
Victoria	22	Canine	[2]
Walker	1	Canine	[2]
Webb	≥ 1	Triatoma	[3]
	2	Canine	[2]
Willacy	≥ 1	Triatoma	[3]
Williamson	≥ 1	Triatoma	[3]
	22	Canine	[2]

Zavala	≥ 1	Triatoma	[3]

References

1. Hanford EJ, Zhan FB, Lu Y, Giordano A (2007) Chagas disease in Texas: Recognizing the significance and implications of evidence in the literature. Social Science and Medicine 65: 60 - 79.

2. Kjos SA, Snowden KF, Craig TM, Lewis B, Ronald N, et al. (2008) Distribution and characterization of canine Chagas disease in Texas. Veterinary Parasitology 152: 249-256.

3. Kjos SA, Snowden KF, Olson JK (2009) Biogeography and *Trypanosoma cruzi* infection prevalence of Chagas disease vectors in texas, USA. Vector-Borne and Zoonotic Diseases 9: 41-50.

4. Pippin WF (1970) The biology and vector capability of *Triatoma sanguisuga texana* (Usinger) and *Triatoma gerstaeckeri* (Stal) compared with Rhodinus (Stal) (Hemiptera: Triatominae). Journal of Medical Entomology 7: 30-45.

5. Lathrop G, Ominsky A (1965) Chagas disease study in a group of individuals bitten by North American Triatomids. USAF School of Aerospace Medicine (AFSC), Brooks AFB, TX, Review 9: 1-5.

6. Kagan I, Norman N, Allain D (1966) Studies on *Trypanosoma cruzi* isolated in the United States: A review. Revista de Biologia Tropical 14: 55-73.

7. Beard CB, Pye G, Steurer FJ, Rodriguez R, Campman R, et al. (2003) Chagas Disease in a domestic transmission cycle in southern Texas, USA. Emerging Infectious Diseases 9: 103-105.

8. Eads R, Trevino H, Campos E (1963) Triatoma (Hemiptera: Reduvidae) infected with *Trypanosoma cruzi* in South Texas wood rat dens. The Southwestern Naturalist 8: 38-42.

9. Burkholder J, Allison T, Kelly V (1980) *Trypanosoma cruzi* (Chagas) (Protozoa: Kinetoplastida) in invertebrate, reservoir, and human hosts of the lower Rio Grande valley of Texas. Journal of Parasitology 66: 305-311.

10. Schuermann JA (1998) Chagas disease in a North Texas Portuguese water dog. Internal memorandum, January 16, Texas Department of Health, Zoonosis Control Division, Austin, TX.

11. Rivera J, Hillis L, Levine B (2004) Reactivation of cardiac Chagas disease in acquired immune deficiency syndrome. American Journal of Cardiology 94: 1102-1103.

12. Cimo P, Luper W, Scouros M (1993) Transfusion-associated Chagas disease in Texas: Report of a case. Texas Medicine 89: 48-50.

13. Yaeger R (1961) The present state of Chagas' disease in the United States. Bulletin of the Tulane University Medical Faculty 21: 9-13.

14. Di Pentima M, Hwang L, Skeeter C, Edwards M (1999) Prevalence of antibody to *Trypanosoma cruzi* in pregnant Hispanic women in Houston. Clinical Infectious Diseases 28: 1281-1285.

15. Leiby D, Rentas F, Nelson K, Stambolis V, Ness P, et al. (2000) Evidence of *Trypanosoma cruzi* infection (Chagas disease) among patients undergoing cardiac surgery. Circulation 102: 2978-2982.

16. Leiby D, Fucci M, Stumpf R (1999) *Trypanosoma cruzi* in a low- to moderate-risk blood donor population: Seroprevalence and possible congenital transmission. Transfusion 39: 310-315.

17. Williams G, Adams L, Yaeger R, McGrath R, Read W, et al. (1977) Naturally occurring trypanosomiasis (Chagas' disease) in dogs. Journal of American Veterinary Medicine Association 171: 171-177.

18. Woody N, DeDianous N, Woody H (1961) American trypanosomiasis II. Current serologic studies on Chagas' disease. Journal of Pediatrics 58: 738-747.

19. Woody N, Hernandez A, Suchow B (1965) American trypanosomiasis III. The incidence of serologically diagnosed Chagas' disease among persons bitten by the insect vector. Journal of Pediatrics 66: 107-109.

20. Betz T (1984) Chagas disease investigation. Texas Preventable Disease News 31: 1-4.
