# Supplementary material for: Chagas Disease Risk in Texas
Source: PLoS Negl Trop Dis. 2010 Oct 5;4(10):e836. doi: 10.1371/journal.pntd.0000836 (PMC2950149; doi:10.1371/journal.pntd.0000836)
Supplement: Table S2 — Species records in the Disease Vectors Database. (0.05 MB PDF) [file pntd.0000836.s008.pdf]

Chagas Disease Risk in Texas

Sahotra Sarkar[1,2,*], Stavana E. Strutz[1], David M. Frank[2], Chissa–Louise Rivaldi[1], Blake Sissel[1], Victor Sánchez–Cordero[3]
1 Section of Integrative Biology, University of Texas, Austin, TX 78712, USA
2 Department of Philosophy, University of Texas, Austin, TX 78712, USA
3 Instituto de Biología, Universidad Nacional Autónoma de México, México City, México 04510
∗ Section of Integrative Biology, University of Texas, Austin, TX 78712, USA; Phone: 1 512 232 3800; FAX: 1 512 471 4806; E-mail: sarkar@mail.utexas.edu

Table S2

Species Records in the Disease Vectors Database (www.diseasevectors.org; [1]). This table is restricted to the seven Texas species (and ignores *T. recurva*). These are the total number of records for these species, and not restricted to Texas.

Species	Number of Records	Number of Post–1960 Records
T. gerstaeckeri	160	142
T. indictiva	9	9
T. lecticularia	32	22
T. neotomae	6	2
T. protracta	32	28
T. rubida	28	22
T. sanguisuga	156	94

References

1. Moffett A, Strutz S, Guda N, González C, Ferro MC, et al. (2009) A global public database of disease vector and reservoir distributions. PLoS Neglected Tropical Diseases 3: e378.
